# Interest and Utility of *MC1R* Testing for Melanoma Risk in Dermatology Patients with a History of Nonmelanoma Skin Cancer

**DOI:** 10.1155/2022/4046554

**Published:** 2022-07-31

**Authors:** Jennifer L. Hay, Erica H. Lee, Stephanie N. Christian, Elizabeth Schofield, Jada G. Hamilton, Ciyu Yang, Bobak Hedayati, Keimya Sadeghi, Mark E. Robson, Allan Halpern, Liying Zhang, Irene Orlow

**Affiliations:** ^1^Memorial Sloan Kettering Cancer Center, New York, NY, USA; ^2^University of Pittsburgh, School of Public Health, Pittsburgh, PA, USA; ^3^University of California, Los Angeles (UCLA), Los Angeles, CA, USA

## Abstract

Public access to genetic information is increasing, and community dermatologists may progressively encounter patients interested in genetic testing for melanoma risk. Clarifying potential utility will help plan for this inevitability. We determined interest and uptake of genetic risk feedback based on melanocortin receptor gene (*MC1R*) variants, immediate (two weeks) responses to risk feedback, and test utility at three months in patients (age ≥ 18, with a history of nonmelanoma skin cancer). Participants (*N* = 50) completed a baseline survey and were invited to consider *MC1R* testing via the study website. Testing interest and uptake were assessed through registration of test decision, request of a saliva test kit, and kit return (all yes/no). Immediate responses to risk feedback included feedback-relevant thoughts, emotions, communication, and information seeking after result receipt; test utility outcomes included family and physician communication and information seeking. Results indicated good retention at both time points (76%; 74%). Half (48%) logged onto the study website, and of these, most (92%) chose testing and (95%) returned a saliva sample. After two weeks, most (94%) had read all the risk feedback information and distress was low (*M* = 8.81, 7–28, SD = 2.23). Many (69%) had talked with their family about the results. By three months, most had spoken with family (92%) and physicians (80%) about skin cancer risk. Physician communication was higher (70%) in those tested versus those not tested (40%, *p* = 0.02). The substantial interest and promising outcomes associated with *MC1R* genetic testing in dermatology patients inform intervention strategies to enhance benefits and minimize risks of skin cancer genetic testing.

## 1. Background

Nonmelanoma skin cancers (NMSCs), predominantly basal cell and squamous cell cancers, are the most common cancers in the United States, and while they are rarely fatal [1], individuals with NMSC are at a two-to-threefold increased risk for developing melanoma, the most fatal form of skin cancer and the most common second primary cancer after diagnosis with NMSC [2, 3]. Despite the heightened risk, prevention and control strategies are underutilized in those with a history of NMSC. For instance, screening has the potential to identify early melanomas, yet most (70%) individuals with an NMSC history did not have a skin examination in the year after they were diagnosed with NMSC [4]. Furthermore, given that ultraviolet radiation (UV) exposure is the primary environmental risk factor for skin cancers— including melanoma—[5–8], many patients continue skin cancer risk behaviors after NMSC diagnosis by indoor tanning [9], not using sunscreen [4, 10, 11], and not protecting themselves from sun exposure [11]. Overall, new methods are needed to heighten risk awareness for skin cancer, especially melanoma, in those with an NMSC history.

Genetic risk feedback based on the type of inherited melanocortin receptor (MC1R) variants carried in an individual is a promising approach to increasing melanoma risk awareness in those with a history of NMSC [12]. MC1R plays an important role in pigmentation, as briefly summarized next. Upon stimulation (e.g., exposure to solar or artificial ultraviolet radiation), binding of alpha-melanocyte-stimulating hormone (*α*-MSH) activates its receptor, MC1R; and this, sequentially, activates adenyl cyclase, increases levels of cAMP in melanocytes, and recruits key controllers of the amounts and proportion of eumelanin and pheomelanin-pigments that can lead to the brown-black or yellow-reddish skin tones, respectively. Polymorphisms in the gene encoding for MC1R (*MC1R*) are very common, with over half of the general population carrying one or more variants that are impactful as they exhibit lower binding capacity or affinity for *α*-MSH (V92M, D84E, D294H) and/or diminished stimulation of intracellular cAMP production in response to *α*-MSH (V60L, R151C, R160W) [23], and are widely reported in association with melanoma risk (referred to as “R” variants in this report) [13] regardless of the individual's lighter or darker skin type [14–16], presumably through the interaction of melanin types (and ratios) with sun exposure and other mechanisms [17, 18]. Because many risk factors for melanoma also increase the risk for NMSC, such as family history, skin type, and sun exposure, carriage of one or more *MC1R “*R” variants likely confers a higher risk for melanoma among patients with NMSC compared to those who are noncarriers. *MC1R* genetic testing and feedback may have utility in raising melanoma and nonmelanoma skin cancer risk awareness and improving communication [19] between patients, family, and physicians about skin cancer risk, ultimately prompting screening and protective behaviors [20].

Given burgeoning public access, the best use of genetic testing by patients rests on developing evidence of any harm, as well as clinical utility, which will shape counseling and education efforts by the medical team to optimize use. One context where this will likely unfold is dermatology care. As such, we examined the interest, uptake, and outcomes of melanoma genetic risk testing. Our specific aims are to examine interest and uptake of *MC1R* testing in individuals with a history of NMSC (Aim I), immediate responses to risk feedback (cognitive, affective, family communication, and information seeking) two weeks after risk feedback receipt (Aim II), and the potential utility of *MC1R* testing (physician and family communication, information seeking) after three months (Aim III). These findings will dictate the feasibility and potential outcomes associated with such testing with individuals who have a history of NMSC to shape future dermatology counseling and education efforts.

## 2. Methods

### 2.1. Participants

Participants were patients recruited in dermatology clinics at an urban tertiary care cancer center. Eligibility criteria included age 18 or older, English fluency, and prior history of NMSC as per medical records. Patients with a history of melanoma were excluded. The study was reviewed and approved by the institutional review board at the institution where the research was conducted.

### 2.2. Procedure

Eligible patients were approached by a trained clinical research coordinator and provided with handouts with basic study information. Patients were consented in the clinic or subsequently by phone based on their preference. Consented participants completed a baseline survey in person, electronically via REDCap with a dedicated study computer, or remotely (by phone with study staff via REDCap). Upon completion of the baseline survey, participants received an invitation letter (via mail, Email, or in person based on their preferred channel for the baseline survey) to consider *MC1R* testing through a secure website. The invitation letter contained participants' personal login information for the secure website.

The website included three educational modules regarding *MC1R* testing adapted from published materials [21, 22] for those with a history of NMSC and a fourth module where participants could opt for, or decline, *MC1R* testing. Participants had up to three months to complete the modules and register a test decision. Those who registered a decision to proceed with testing received a prelabeled saliva collection kit (Oragene OGR-500, DNAGenotek Inc.) and instructions, which allowed them to provide a sample in a mailer envelope addressed, and postage-paid back to the research facility. Samples received were recorded in a study tracking log and then hand-delivered to the CLIA-certified Diagnostic Molecular Genetics (DMG) Laboratory at MSK for Sanger sequencing of the *MC1R* gene.

The entire *MC1R* coding region was sequenced. For each specimen, a report included information on the *MC1R* status, indicating carriage or absence of polymorphisms previously reported to convey a *higher than average risk for developing melanoma* compared to the general healthy population [13, 14, 16]. Specifically, participants were reported as carriers of one or more *MC1R* “R” alleles when any of the following “R” variant alleles were present: V60L, D84E, V92M, R142H, R151C, I155T, R160W, R163Q, D294H. When no variants or only other nonrisk *MC1R* variants were detected, the participants were considered as non- “R” carriers. In sum, the *MC1R* status classified participants with one or more *MC1R* “R” variants into “higher than average risk,” and those with no *MC1R* “R” variants (with or without nonrisk “r” variants) at “average risk”—compared to other NMSC patients [19, 23].

Two weeks after risk feedback results were mailed, all tested participants received a telephone call, during which they completed an assessment of immediate responses to testing and were given the opportunity to field questions about their results. Participants completed this survey by phone or electronically via REDCap. Three months after baseline, all participants were similarly reached to complete outcome assessments. Participants who completed the baseline survey received $25, the website modules up to $15 ($5/module), the two-week survey $20, and the three-month follow-up survey $20. Participants were not incentivized for undergoing testing.

### 2.3. Measures Battery

#### 2.3.1. Outcome Measures

For Aim I, we assessed study interest and uptake of testing. Registration of a test decision (yes/no), request of a saliva test kit (yes/no), and return of a test kit (yes/no) comprised assessments of study interest and uptake.

For Aim II, we assessed cognitive, affective, family communication, and information seeking from the two-week survey. These were operationalized as to how much of the test results participants reported reading on a five-point Likert-type scale from “none” to “all” of the information. Perceived believability and clarity of the test results were assessed using two seven-point Likert-type items from “strongly disagree” to “strongly agree.” The frequency of thinking about test results was assessed using seven-point Likert-type items from “never” to “all the time.” We assessed emotional responses to receiving genetic test results (i.e., feeling nervous, relieved, regret, afraid, hopeful, confused, and determined to change behavior) using seven items answered on seven-point Likert-type scales from “not at all” to “a great deal”; these items were adapted from the Multiplex study assessing responses to genetic susceptibility test results [24]. Cancer-related distress was assessed using the Impact of Events-Revised Intrusive thoughts subscale, in which distress was treated as a summed score [25] based on seven items on separate five-point scales (“not at all” to “extremely”). This subscale is widely used with good internal and test-retest reliability and a good ability to distinguish those with cancer distress [26, 27]. Finally, we assessed whether participants had talked with their families about their test results or genetic testing (yes/no, respectively) and whether they had engaged in information seeking about skin cancer or genetic testing (yes/no, respectively) since the study onset.

For Aim III, we examined data from the three-month survey regarding whether or not participants had talked to their family members about cancer risk or skin cancer risk, respectively, since study onset (two four-point Likert scales, “not at all” to “a lot”); whether they had, or planned to, talk with their family about skin cancer genetic testing (yes/no, respectively); and whether they had communicated with their doctor about skin cancer risk since study onset (four-point Likert scale, “not at all” to “a lot”) using a measure from prior genetic communication research [20, 28–30]. To assess information seeking, we asked participants whether they had sought information about skin cancer (yes/no) or genetic testing (yes/no) since the beginning of the study.

#### 2.3.2. Covariates

We examined demographics, skin cancer risk factors, and psychosocial factors. The baseline survey assessed ethnicity, race, sex, educational attainment, age, birth country, marital status, employment status, and income. Skin cancer risk factors were also included: history of cancer other than NMSC, family history of skin cancer, and skin type (burnability, tannability, and lifetime number of sunburns) [31].

Psychosocial factors included three items assessing the perceived risk of developing additional skin cancers [32], including the personal likelihood of getting skin cancer again (likely/unlikely, absolute likelihood with seven verbal categories, and comparison with others with five categories, on separate scales including “do not know”); [33] and frequency of worry about getting skin cancer assessed with one item (four-point scale; “rarely or never” to “all the time”) and concern about the possibility of getting skin cancer again with one item (four-point scale; “not at all concerned” to “very concerned”). Worry and concern items were drawn from Lerman and colleagues [34]. We also assessed perceived level of importance to know more about how their genes can affect their chances of developing health conditions (seven-point scale, “not at all important” to “very important”) [20].

### 2.4. Statistical Approach

Participant baseline characteristics were reported overall. We examined multiple dimensions of testing, including the rate of logging onto the study website described in their invitation letter, registering a decision to undergo testing that involves requesting a mailed test kit, and returning their kit by mail with a saliva sample to undergo testing. Frequencies were calculated for website logon (yes versus no), requesting a test kit (yes versus no), and providing a saliva sample for *MC1R* testing (yes versus no). We evaluated crude associations between baseline covariates (demographics, skin cancer risk factors, psychosocial factors) and test completion (yes/no) using a series of logistic regression models. Two-week outcomes (cognitive, affective, family communication measures, information seeking) were reported for test completers. Three-month outcomes (physician and family communication, information seeking) were reported for all participants. Type I error rates were set at 0.05, and statistical analyses were conducted in SAS version 9.4 (Cary, NC).

## 3. Results

We approached 138 eligible patients, and of these, 53 patients consented to study participation. Of those 85 who did not consent, the most frequent reason was not having time to participate (*n* = 51 patients). Of the 53 who consented, 50 completed the baseline assessment. Retention at the two-week assessment among those who returned a kit was very good (76%), as was retention at the three-month assessment (*n* = 37, 74%). Participant (*n* = 50) characteristics are outlined in Table 1.

To address Aim I, we examined the interest and uptake of *MC1R* testing in this sample of NMSC patients. About half (*n* = 24; 48%) logged onto the study website, and of those, most (*n* = 22/24, 92%) registered a decision to undergo testing and thus received a saliva kit. Most of these (*n* = 21/22, 95%) returned their saliva sample for genetic testing and are referred to as “test completers.” Most (19/21, 91%) carried one or higher risk “R” alleles (90.5%); one participant had other nonrisk variants (“r”), and one had no “R” or “r” variants. Twenty-nine participants either did not log on to the study website (*n* = 26), logged on and did not register a decision to test (*n* = 2), or requested a kit but did not return (*n* = 1), and are all referred to as “noncompleters.” None of the baseline demographic, skin cancer risk, or psychosocial factors were associated with returning a sample for genetic testing.

For Aim II, we assessed immediate responses to risk feedback among test completers. Of the 21 completers, 16 (76%) completed this assessment after receiving their test results, and most of these reported reading all the risk feedback information provided (*n* = 15, 94%). Overall, the results were clearly understood (*M* = 5.75, SD = 1.69, 1–7 scale), and the results were believable (*M* = 6.88, SD = 0.34, 1–7 scale). Emotional reactions to test results were unremarkable, and none reported testing regret. Participants responded, on average, near the scale mid-point for determination to change behavior (*M* = 4.38, SD = 2.53, 1–7 scale). Most had talked to their family about genetic testing for skin cancer in general (*n* = 14, 88%) and what their personal results meant for their family (*n* = 11, 69%). Half (*n* = 8, 50%) had sought information on skin cancer, and some (*n* = 3, 19%) sought information on genetic testing since the beginning of the study. Descriptive statistics are shown in Table 2.

By three-month follow-up, most participants (including testing completers and noncompleters) had spoken with family members about both cancer risk in general (*n* = 30, 81%) and skin cancer risk (*n* = 34, 92%), and had spoken with their doctor about skin cancer risk (*n* = 28, 80%). However, less than a quarter had spoken to their family members about genetic testing (*n* = 8, 22%), and of those who had not spoken about genetic testing, less than a third planned to (*n* = 8, 30%). Half of the participants reported that they had sought information about skin cancer since the beginning of the study (*n* = 18, 50%), but few (*n* = 4, 11%) had sought information on genetic testing. Test completers had a higher rate of family communication about genetic testing than those who did not test (35% versus 6%, *p*=0.05). Communication with a doctor varied by testing status, where only 40% of noncompleters reported having spoken some or a lot, compared to 70% of test completers (*p*=0.02). Three-month follow-up results are presented in Table 3.

## 4. Discussion and Conclusion

### 4.1. Discussion

Access to genetic information regarding health risks will be increasingly available to the general public in the coming years. Thus, understanding the potential utility of such information will help physicians and other healthcare providers anticipate and plan for this inevitability and to further shape educational efforts to enhance benefits and minimize risks associated with the use of genetic health information in their patients. Dermatologists may find an increasing proportion of patients, especially those with skin cancer risk factors, who express interest in genetic testing or who have received direct-to-consumer genetic information that they need help interpreting in view of their skin cancer risks. In the current study, we offered testing for *MCIR,* a well-characterized gene of moderate penetrance that is highly polymorphic, for which several variants have been well studied in relation to melanoma risk, to a sample of individuals with a history of NMSC. We found moderate testing uptake, with most who expressed interest following through with providing a biospecimen for genetic testing. We also found high comprehension and low distress associated with receipt of test results and some increase in family and physician communication as well as information seeking about skin cancer and genetic testing among those who pursued testing. These findings are reassuring given that such risk information is provided in the direct-to-consumer and consumer-directed genetic testing settings in which genetic counseling is generally not required. [35] Results suggest that communication and information seeking are important positive outcomes of testing that may benefit patients and the entire family system. Patients may perceive testing as an opportunity to understand and appreciate their risk and raise it as a topic of discussion with family and physicians, which could be a useful patient-driven aspect of personalized medicine.

The use of *MC1R* genetic testing to promote skin cancer prevention and early detection behaviors is developing to date and has been examined in general population samples outside of the dermatology setting. There is meta-analytic support that the provision of higher risk personalized genetic risk feedback motivates health behavior change [36], and work conducted by us and others in the general population indicates that individuals are quite interested in *MC1R* genetic testing and are motivated to use it to make skin health decisions. [22, 37] In these studies and others, the provision of MC1R and similar melanoma genetic risk test results lead to behavioral activation but not worry and promote increased use of sun protection and skin screening, as well as reductions in sunburn in individuals who received the feedback. Further, individuals who receive average risk feedback do not increase risk behaviors such as tanning [38–40]. Current genetic testing technology allows for low-cost comprehensive genomic assessment, and *MC1R* is often evaluated as part of direct-to-consumer genetic tests. Accordingly, extending investigation from the general population into clinical samples, such as dermatology patients, is a promising area for further investigation.

In the current study, we found that study participants had high levels of the perceived risk of developing another skin cancer and were receptive to genetic information, with over half reporting that it was important to learn more about genes and health. Almost half of the participants (48%) logged onto the study website to learn more about *MC1R* testing, and most of these individuals registered a decision to pursue it and followed through with providing a saliva sample. These findings are quite comparable to testing uptake rates in a larger study conducted in the general population, where 46% logged onto the study website and most followed through with providing a saliva sample [22]. The current study shows high levels of interest in skin cancer information, including genetics, which translates into robust rates of uptake of genetic testing when offered.

We examined the responses of those who completed testing two weeks after the result receipt. Findings indicate high levels of reading the entire feedback packet, high comprehension, and satisfaction with the information received, low distress, and predominance of positive emotional outcomes and minimal negative emotional outcomes. Family communication and information seeking all show trends in line with the fact that receipt of genetic information is on balance, informative, interesting, and actionable to patients in terms of their discussions of results across their family systems. The impact on behavioral intentions, while moderate, falls into two modal responses—high intentions to change behaviors and low intentions to change behaviors. While these were small samples, the impact on behaviors may be based on time of year, history of prior sun protection behaviors (which may already be high), or preferred behaviors while outside. Future work examining the impact of skin cancer genetic information on behavior change must consider what behaviors require change and whether the receipt of genetic information requires combination with other intervention components to increase and maintain motivation to change behaviors.

At three months, there were also promising indications of utility, with 35% of those who underwent *MC1R* testing reporting family communication about genetic testing compared to only 6% of those who did not test and 70% of those who underwent *MC1R* testing reporting physician communication about skin cancer risk compared to 40% of those who did not test. By three-month follow-up, information seeking about skin cancer was high, and most participants had spoken with family members about both cancer risk in general and skin cancer risk.

### 4.2. Study Limitations

Study limitations include the fact that this patient sample was clinic-based; examination of more generalizable samples of individuals with a history of NMSC with a range of ages outside of the clinic setting will be imperative to confirm and expand on these findings, especially since test completion may vary by socioeconomic status and health literacy. Our recruitment rate among eligible patients (38%) was low and may indicate lower levels of interest in *MC1R* testing among the broader population of dermatology patients, although this was not explicitly mentioned by study refusers. Additionally, the sample assessed was small, and thus we were limited in our ability to conduct inferential statistics. We did not have access to chart information, such as patient histories of specific skin cancer types and current or past prevention or intervention therapies; interest and use of *MC1R* testing may differ across these factors and are critical areas for future investigation. Finally, the study was not designed to look at individuals with high levels of nonadherence to sun protection; thus, examination of whether genetic testing feedback motivates behavior change will need to wait for future studies that target those with low levels of sun protection and adequate numbers of those who receive diverse test results (higher risk; average risk) given that behavioral activation may differ by type of risk feedback received [36].

## 5. Conclusion

We report promising outcomes associated with *MC1R* genetic testing in individuals with a history of NMSC. The level of interest, positive responses to risk feedback, as well as the utility of testing all represent an encouraging picture for the use of such a tool for individuals who have melanoma risk factors, such as a personal history of NMSC. Studies examining behavioral utility and extension to community and population-based samples of patients are warranted.

### 5.1. Clinical Implications

Dermatologists should anticipate that patients with skin cancer risk factors or heightened skin cancer concerns may express interest in genetic testing or pursue their own direct-to-consumer genetic information about their risk of developing melanoma. Patients may perceive testing as an opportunity to understand and engage around their risk and raise it as a topic of discussion with family and physicians, which could be a useful patient-driven aspect of personalized medicine. Given that *MC1R* also predisposes to NMSC [27], patients may be interested in the implications for genetic risk for a range of skin cancer types. Physicians may find it useful to enhance their educational messaging regarding the benefits and limitations of genetic testing in the context of melanoma risk and to help their patients to interpret risk feedback results in the context of other skin cancer and other cancer risks, as well as their risk behavior.

## Figures and Tables

**Table 1:** Participant baseline characteristics, overall, and saliva sample return status.

Characteristic	Group	All (*n* = 50)	Noncompleters^*∗∗*^ (*n* = 29)	Completers (*n* = 21)	*p*-value
*DEMOGRAPHICS*					
Gender	Female	28 (56%)	14 (48%)	14 (67%)	0.20
Education	No college degree	9 (18%)	6 (21%)	3 (14%)	0.74
	College graduate	18 (36%)	8 (28%)	10 (48%)	
	Graduate degree	23 (46%)	15 (52%)	8 (38%)	
Age (years)	Mean (SD) [35–78]	63.6 (10.7)	62.9 (10.8)	64.6 (10.8)	0.11
Born outside US	Yes	8 (16%)	6 (21%)	2 (10%)	0.29
Marital Status (*n* = 49)	Single	6 (12%)	4 (14%)	2 (10%)	0.51
	Married/Cohabitating	39 (80%)	23 (82%)	16 (76%)	
	Divorced/Separated	3 (6%)	1 (4%)	2 (10%)	
	Widowed	1 (2%)	0 (0%)	1 (5%)	
Employed	Yes	26 (52%)	18 (62%)	8 (38%)	0.09

*SKIN CANCER RISK FACTORS*					
Other Cancer	Yes	16 (32%)	8 (28%)	8 (38%)	0.43
Family History (*n* = 48)	Yes	31 (65%)	17 (61%)	14 (70%)	0.51
Burnability (*n* = 48)	Severe	1 (2%)	0 (0%)	1 (5%)	0.13
	Painful/peeling	20 (42%)	10 (36%)	10 (50%)	
	Mild/tan	27 (56%)	18 (64%)	9 (45%)	
Tannability (*n* = 45)	Very brown & tanned	6 (13%)	3 (12%)	3 (15%)	0.26
	Moderately tanned	13 (29%)	10 (40%)	3 (15%)	
	Mild tan/some peeling	16 (36%)	8 (32%)	8 (40%)	
	Freckle/no tan	10 (22%)	4 (16%)	6 (30%)	
Lifetime number of sunburns (*n* = 48)	0	10 (21%)	7 (25%)	3 (15%)	0.48
	1–2	14 (29%)	9 (32%)	5 (25%)	
	3–5	11 (23%)	5 (18%)	6 (30%)	
	6–9	6 (13%)	3 (11%)	3 (15%)	
	10–14	4 (8%)	2 (7%)	2 (10%)	
	15–19	1 (2%)	1 (4%)	0 (0%)	
	20 or more	2 (4%)	1 (4%)	1 (5%)	

*PSYCHOSOCIAL*					
Perceived risk (absolute)	Unlikely to get SC	4 (8%)	3 (10%)	1 (5%)	0.67
	Likely to get SC	40 (80%)	22 (76%)	18 (86%)	
	No idea	6 (12%)	4 (14%)	2 (10%)	
Perceived risk (absolute)	1 No chance	3 (6%)	1 (3%)	2 (10%)	0.29
	2 Very Unlikely	1 (2%)	1 (3%)	0 (0%)	
	3 Unlikely	3 (6%)	3 (10%)	0 (0%)	
	4 Moderate Chance	13 (26%)	9 (31%)	4 (19%)	
	5 Likely	17 (34%)	11 (38%)	6 (29%)	
	6 Very Likely	12 (24%)	4 (14%)	8 (38%)	
	7 Certain to happen	1 (2%)	0 (0%)	1 (5%)	
Perc. risk (abs.)	Mean (SD) [1–7]	4.60 (1.36)	4.38 (1.18)	4.90 (1.55)	0.18
Perceived risk (comparative) (*n* = 49)	1 Well below average	0 (0%)	0 (0%)	0 (0%)	0.11
	2 Below average	2 (4%)	2 (7%)	0 (0%)	
	3 Average	16 (33%)	10 (36%)	6 (29%)	
	4 Above average	27 (55%)	15 (54%)	12 (57%)	
	5 Well above average	4 (8%)	1 (4%)	3 (14%)	
Perc. risk (comp.)	Mean (SD) [1–5]	3.67 (0.69)	3.54 (0.69)	3.86 (0.65)	0.11
Worry about SC	1 Rarely or never	28 (56%)	17 (59%)	11 (52%)	0.81
	2 Sometimes	11 (22%)	5 (17%)	6 (29%)	
	3 Often	10 (20%)	7 (24%)	3 (14%)	
	4 All the time	1 (2%)	0 (0%)	1 (5%)	
Worry about SC	Mean (SD) [1–4]	1.68 (0.87)	1.66 (0.86)	1.71 (0.90)	0.81
Concern about SC	1 Not at all concerned	24 (48%)	15 (52%)	9 (43%)	0.84
	2	10 (20%)	4 (14%)	6 (29%)	
	3	11 (22%)	6 (21%)	5 (24%)	
	4 Very concerned	5 (10%)	4 (14%)	1 (5%)	
Concern about SC	Mean (SD) [1–4]	1.94 (1.06)	1.97 (1.15)	1.90 (0.94)	0.84
Importance of learning about your genes	1 Not at all important	2 (4%)	0 (0%)	2 (10%)	0.25
	2	1 (2%)	0 (0%)	1 (5%)	
	3	0 (0%)	0 (0%)	0 (0%)	
	4 Neither unimportant nor important	4 (8%)	3 (10%)	1 (5%)	
	5	5 (10%)	4 (14%)	1 (5%)	
	6	8 (16%)	4 (14%)	4 (19%)	
	7 Very important	30 (60%)	18 (62%)	12 (57%)	
Importance	Mean (SD) [1–7]	6.06 (1.54)	6.28 (1.07)	5.76 (2.02)	0.25

*INFORMATION SEEKING*					
SC	Yes	44 (88%)	26 (90%)	18 (86%)	0.67
Genetic testing	Yes	13 (26%)	9 (31%)	4 (19%)	0.34

^*∗*^Note that *p*-values for continuous variables (e.g., age) are based on independent samples *t*-tests; purely categorical variables (e.g., gender and marital status) are based on the Chi-square test; and ordinal variables (e.g., education or number of lifetime sunburns) are based on Mantel-Haenszel Chi-square test. SC denotes skin cancer. “^*∗∗*^Noncompleters” includes the 29 participants who either did not log on to the study website (*n* = 26), logged on and did not register a decision to test (*n* = 2) or requested a kit but did not return it (*n* = 1).

**Table 2:** Comprehension, distress, and communication outcomes two weeks after risk feedback receipt (*n* = 16).

Characteristic	Mean (SD)	Characteristic	*n* (%)
Believability [1–7]	6.88 (0.34)	Family Comm.—genetic testing, yes	14 (88%)
Clarity [1–7]	5.75 (1.69)	Family Comm.—test results, yes	11 (69%)
Thought about [1–7]	3.81 (1.33)	Info. seeking—skin cancer, yes	8 (50%)
Nervous [1–7]	1.75 (1.61)	Info. seeking—genetic testing, yes	3 (19%)
Relieved [1–7]	3.06 (2.29)	Read all of the information, all	15 (94%)
Regret [1–7]	1.00 (0.00)		
Afraid [1–7]	1.50 (1.10)		
Hopeful [1–7]	4.63 (2.36)		
Confused [1–7]	1.94 (1.61)		
Determined to change [1–7]	4.38 (2.53)		
Distress, Sum [7–28]	8.81 (2.23)		

^†^Ranges indicate potential, not necessarily observed, minimum, and maximum. ^‡^Family Comm denotes family communication; Info. Seeking denotes information seeking.

**Table 3:** Communication and information seeking outcomes at three months, by saliva sample return status.

Characteristic	All (*n* = 37)	Noncompleters (*n* = 17)	Completers (*n* = 20)	*p*-value
*Family Comm. cancer risk*				
Not at all	7 (19%)	5 (29%)	2 (10%)	0.64
A little	12 (32%)	4 (24%)	8 (40%)	
Some	13 (35%)	5 (29%)	8 (40%)	
A lot	5 (14%)	3 (18%)	2 (10%)	

*Family Comm. skin cancer risk*				
Not at all	3 (8%)	2 (12%)	1 (5%)	0.26
A little	16 (43%)	8 (47%)	8 (40%)	
Some	14 (38%)	6 (35%)	8 (40%)	
A lot	4 (11%)	1 (6%)	3 (15%)	

*Comm. with doctor*				
Not at all	7 (20%)	6 (40%)	1 (5%)	0.02
A little	8 (23%)	3 (20%)	5 (25%)	
Some	14 (40%)	5 (33%)	9 (45%)	
A lot	6 (17%)	1 (7%)	5 (25%)	

*Family Comm. genetic testing*				
No	28 (78%)	15 (94%)	13 (65%)	0.05
Yes	8 (22%)	1 (6%)	7 (35%)	

*Family Comm. (planned)—genetic testing*				
No	19 (70%)	11 (79%)	8 (62%)	0.42
Yes	8 (30%)	3 (21%)	5 (38%)	

*Information Seeking—skin cance*r				
No	18 (50%)	10 (63%)	8 (40%)	0.18
Yes	18 (50%)	6 (38%)	12 (60%)	

*Information Seeking—genetic testing*				
No	32 (89%)	15 (94%)	17 (85%)	0.41
Yes	4 (11%)	1 (6%)	3 (15%)	

^†^Comm denotes communication. ^*∗*^*p*-values based on Mantel-Haenszel Chi-square tests for ordinal variables and exact Chi-square tests for dichotomous (yes/no) variables.

## Data Availability

The data that support the findings of this study are available from the corresponding author upon reasonable request.
